# Supplementary material for: Rapid turnover of life-cycle-related genes in the brown algae
Source: Genome Biol. 2019 Feb 14;20:35. doi: 10.1186/s13059-019-1630-6 (PMC6374913; doi:10.1186/s13059-019-1630-6)
Supplement: Supplementary file 3 — Protein alignments, corresponding Gblocks html file and CDS sequences for the lineage conserved gametophyte-biased genes. (ZIP 2109 kb) [file 13059_2019_1630_MOESM3_ESM.zip › Con_GA_alignments/Ec-11_005490.aa_ali.fasta-gb.htm]

Ec-11\_005490.aa.fasta.trans.aa\_ali.fasta


## Gblocks 0.91b Results

Processed file: **Ec-11\_005490.aa.fasta.trans.aa\_ali.fasta**  
Number of sequences: **2**  
Alignment assumed to be: **Protein**  
New number of positions: **297** (selected positions are underlined in blue)

```
                         10        20        30        40        50        60
                 =========+=========+=========+=========+=========+=========+
Ec-11_005490     ------------------------------------------------------------
MSTRG.39278.1    PVHSTKAAAAGYCWFDSRFGGAMGHFPMNGIASHARGEDENPCDANGVNHEKDFTSVAAP
                                                                             


                         70        80        90       100       110       120
                 =========+=========+=========+=========+=========+=========+
Ec-11_005490     -------------MTCVISLGCQ---VRRERDSDPIKTELMMRRGQLLVRFAREGRLRDM
MSTRG.39278.1    SATVTWGLHQSRIFAMTETTDAQLRRIKRERDTDPGKTGRTMRRGQLLVRFAREGRLRGI
                                             ################################


                        130       140       150       160       170       180
                 =========+=========+=========+=========+=========+=========+
Ec-11_005490     DQVSRSCPRGEMLAWFTVRMFKEACAHNHLQTMRFMVDHGLDVEFHAVLEAVHWLVDGCD
MSTRG.39278.1    DQIVSRCPREEMLAWFVVRMFKGACLHNRLPTIVYMVDRGLDVEFHPVLEALHWVVEGCD
                 ############################################################


                        190       200       210       220       230       240
                 =========+=========+=========+=========+=========+=========+
Ec-11_005490     EEQDGIDVVPAIDFLVTEAGMPPDVQRKSDFRTPLHVAVRRGLFEAARRLIELGADVNAV
MSTRG.39278.1    EEQDGIDVVPTLNFLVTVAGMRPDQQRRSDFHTPLHVAIQRGLFEAARRLIELGADINAV
                 ############################################################


                        250       260       270       280       290       300
                 =========+=========+=========+=========+=========+=========+
Ec-11_005490     AKMAPLGNAKGDVMPLTLAEEPAET----------------D-GFRATDDTSPEAGDDVA
MSTRG.39278.1    A--------KEDAMPLTLSEQPVAQDESCAPAVTPGTGEGCDGGLVGETLTSPPLPPPTT
                 #        #############                     #################


                        310       320       330       340       350       360
                 =========+=========+=========+=========+=========+=========+
Ec-11_005490     TKGKAAEIAAGAAAVNRYHLTRNDGARDEQGVREEDHDVERMSLAAERGRQCGEETSHVG
MSTRG.39278.1    TTTTTGVMKALPNQSFQVIHTSGGGEVVEKSERE-GRDQEKAFVSGVVGKTREEGGGGVG
                 ##########          ##############   #######################


                        370       380       390       400       410       420
                 =========+=========+=========+=========+=========+=========+
Ec-11_005490     GVGARSPSPSSRSLCRRRWSRVMASRARICEILVERGARRGWRRAPSEEQVALDKKY-AV
MSTRG.39278.1    GLAP-QPSSSSSTINSRRRMRVAVSRARICGLLVERGARRTWRRGRSEAQVALDEKYRLL
                 #     ###################################################   


                        430
                 =========+=====
Ec-11_005490     LLEGDDGRNFCTFSG
MSTRG.39278.1    LLEGDGGRNLSTFNG
                 ###############
```

```
Parameters used
Minimum Number Of Sequences For A Conserved Position: 2
Minimum Number Of Sequences For A Flanking Position: 2
Maximum Number Of Contiguous Nonconserved Positions: 8
Minimum Length Of A Block: 10
Allowed Gap Positions: None
Use Similarity Matrices: Yes
```

```
Flank positions of the 7 selected block(s)
Flanks: [89  241]  [250  262]  [284  310]  [321  334]  [338  361]  [367  417]  [421  435]  

New number of positions in Ec-11_005490.aa.fasta.trans.aa_ali.fasta-gb:  297  (68% of the original 435 positions)
```
